# Searching for ancient balanced polymorphisms shared between Neanderthals and Modern Humans

**DOI:** 10.1590/1678-4685-GMB-2017-0308

**Published:** 2018

**Authors:** Lucas Henriques Viscardi, Vanessa Rodrigues Paixão-Côrtes, David Comas, Francisco Mauro Salzano, Diego Rovaris, Claiton Dotto Bau, Carlos Eduardo G. Amorim, Maria Cátira Bortolini

**Affiliations:** 1 Departamento de Genética, Instituto de Biociências, Universidade Federal do Rio Grande do Sul, Porto Alegre, RS, Brazil; 2 Departamento de Biologia, Instituto de Biologia, Universidade Federal da Bahia, Salvador, BA Brazil; 3 Institut de Biologia Evolutiva (CSIC-UPF), Departament de Ciències Experimentals i de LaSalut, Universitat Pompeu Fabra, Barcelona, Spain; 4 Department of Biological Sciences, Columbia University, New York, NY, U.S.A.; 5 Department of Ecology and Evolution, Stony Brook University, Stony Brook, NY, U.S.A.

**Keywords:** Human behavior, evolution, balancing selection, immune genes, behavioral genes

## Abstract

Hominin evolution is characterized by adaptive solutions often rooted in behavioral and cognitive changes. If balancing selection had an important and long-lasting impact on the evolution of these traits, it can be hypothesized that genes associated with them should carry an excess of shared polymorphisms (trans- SNPs) across recent *Homo* species. In this study, we investigate the role of balancing selection in human evolution using available exomes from modern (*Homo sapiens*) and archaic humans (*H. neanderthalensis* and Denisovan) for an excess of trans-SNP in two gene sets: one associated with the immune system (IMMS) and another one with behavioral system (BEHS). We identified a significant excess of trans-SNPs in IMMS (N=547), of which six of these located within genes previously associated with schizophrenia. No excess of trans-SNPs was found in BEHS, but five genes in this system harbor potential signals for balancing selection and are associated with psychiatric or neurodevelopmental disorders. Our approach evidenced recent *Homo* trans-SNPs that have been previously implicated in psychiatric diseases such as schizophrenia, suggesting that a genetic repertoire common to the immune and behavioral systems could have been maintained by balancing selection starting before the split between archaic and modern humans.

## Introduction

Many of the fundamental processes at the core of complex human behavior and cognitive abilities, including sensory processing, recognition of self and others, emotions, motivation, learning, memory, attention, vocalization and speech processing, executive function, as well as neural development, may be characterized by at least some degree of heritability (Vallender, 2011; Gilissen *et al.*, 2014; Vissers *et al.*, 2015; Johnson *et al.*, 2016). Assuming that genes are partly responsible for these phenotypes, genetic variation between and within species can be expected to give rise to the large behavioral repertoire observed in nature (Bendesky and Bargmann, 2011). Furthermore, the diversity of these traits can be expected to be shaped by the fundamental forces of microevolution, including genetic drift, directional natural selection (either positive or negative), and balancing selection.

While directional selection tends to reduce variability close to the selected site (Lachance and Tishkoff, 2013), balancing selection results in the persistence of variation in the population or species, even in the face of loss due to drift, leading to an excess of polymorphisms with intermediate frequencies (Nielsen *et al.*, 2009) and increasing genetic diversity around the site of selection (Charlesworth, 2006; Fijarczyk and Babik, 2015). Balancing selection can result from different processes, such as heterozygote advantage (overdominance), negative frequency-dependent selection, and heterogeneity in selective advantage across time or space – all possibly acting in changing environments requiring a fast rate of adaptation (Boon *et al.*, 2007; Wolf *et al.*, 2007; Bendesky and Bargmann, 2011; Pruitt and Riechert, 2011; Schaschl *et al.*, 2015; Taub and Page, 2016).

While many cases of directional selection have been reported in the literature, only a handful of examples of balancing selection have been described. This may be due to several factors. On the one hand, balancing selection may be a transient state, leaving marks so subtle that their detection may be difficult using current tests, leading to a large number of false negatives (Fijarczyk and Babik, 2015). On the other hand, most methods rely on the fact that balancing selection will lead to a decreased inter-population diversity. In populations that diverged not long ago, or that are experiencing some level of admixture, however, this pattern will be found at most neutral loci, thereby leading to a large number of false positives. One way of circumventing this issue is by comparison of different species, considering ancient balancing selection, in which most loci should indicate moderate to high divergence, and thus the number of false positives is expected to be smaller.

One of the best-studied targets of balancing selection is the major histocompatibility complex (MHC), which includes many examples of long-term maintenance of trans-species polymorphisms (trans-SNPs), *i.e.* ancient polymorphisms that survived in derived taxa (Takahata and Nei, 1990; Clark, 1997; Grimsley *et al.*, 1998; Ségurel *et al.*, 2013; Azevedo *et al.*, 2015). The study of these trans-SNPs has revealed a common theme, where individuals heterozygous for genes with key roles in the immune system seem to be more effective in their defense against pathogens, while at the same time presenting only a moderate inflammatory response (Cagliani *et al.*, 2008; Leffler *et al.*, 2013; Azevedo *et al.*, 2015; Teixeira *et al.*, 2015).

Because of the expected loss of shared polymorphic sites over time due to genetic drift, polymorphisms shared between species that diverged a long time ago are rare under neutrality (Clark, 1997). Trans-SNPs common to species separated by a relatively deep evolutionary split, and without recent admixture, are therefore probably adaptive and maintained by balancing selection. Examples of such adaptive trans-SNPs were reported by Leffler *et al.* (2013) and Teixeira *et al.* (2015) in their comparison between humans and the *Pan* genus, which are thought to have diverged about 8 million years ago (Moorjani *et al.*, 2016). In more recently-diverged species, the presence of trans-SNPs must be interpreted with greater caution. For instance, for humans, which have an estimated effective population size (N*e*) of ~10,000 individuals, 1% of neutral trans-SNPs will be preserved in the genome even after 53,000 generations (~1,6 million years) (Clark, 1997). Assuming that the split between *Homo sapiens* and *Homo neanderthalensis* occurred around 400,000-275,000 years ago (Endicott *et al.*, 2010; Prüfer *et al.*, 2014), some trans-SNPs occurring in these species are expected to be neutral and occur due to stochastic events. Additionally, the retention of ancestral polymorphisms can be due to introgression, the exchange of genetic material between different species due to hybridization (Fijarczyk and Babik, 2015). This has been described for the hybridization between archaic humans (including *H. neanderthalensis* and the closely related Denisovans) and some modern human populations (Green *et al.*, 2010; Reich *et al.*, 2010; Meyer *et al.*, 2012).

Despite these difficulties, the investigation of trans-SNPs in the genus *Homo* is an exciting research goal. Hominin evolution is characterized by adaptive solutions rooted in behavioral and cognitive changes. For instance, creative thinking promotes change from prevailing modes of thought or expression, a change that can be associated with fitness gain (Nettle, 2006). In addition to the benefits of change over time, there are adaptive advantages to the parallel maintenance of different behavioral strategies within a species (Sih *et al.*, 2004; Korte *et al.*, 2005; Cagliani *et al.*, 2009; Taub and Page, 2016). Assuming a genetic basis for these behavioral strategies, their parallel persistence can be seen as the result of balancing selection. In support of the idea for balancing selection, there have been several reports of polymorphisms in genes with known roles in modulating complex behavior in modern human and other mammals, which have likely persisted through balancing selection (Cagliani *et al.*, 2009; Schaschl *et al.*, 2015; Taub and Page, 2016).

Expanding on this idea, it is of great interest to investigate the role of balancing selection in the evolution of hominin, including human, behavior on a greater scale. If balancing selection has indeed had an important and long-lasting impact on the evolution of behavior in hominins, it can be hypothesized that genes associated with behavior should carry an excess of trans-SNPs across hominin species. Based on this hypothesis, we investigated the role of balancing selection in the evolution of behavior in hominins by studying the pattern of trans-SNPs in genes relevant to these traits in Neanderthals and modern humans.

Importantly, as previously mentioned, many available methods used to detect balancing selection were originally designed to target balancing selection operating over more than 4N*e* generations (Clark, 1997). Due to the relatively recent split between modern humans and Neanderthals, these methods are ineffective in detecting balancing selection when studying these two species. To overcome this limitation, we implemented an approach that enables the identification of an excess of trans-SNPs in groups of genes of interest in comparison to the exome background (*i.e.* the null distribution), which serves as a control for demographic effects, while we added control for gene length biases, GC content (and thus indirectly mutation rate), number of polymorphisms per gene and background selection. Using this approach, we were able to identify polymorphisms shared between modern humans and Neanderthals, many of which located in genes related to immunology and a few in genes playing a potential role in behavior, including genes that may contribute to personality traits and psychiatric disorders.

## Material and Methods

### Defining gene sets

To find genes underlying immune and behavioral systems, we defined two target gene sets, which we named IMMS (genes related to the immune system) and BEHS (genes related to behavior). We populated these gene sets by searching the AmiGO database (http://amigo.geneontology.org/cgi-bin/amigo/browse.cgi) using GeneOntology terms directly related to immune system (for IMMS) and behavior (for BEHS; supplementary material Table S1; http://amigo.geneontology.org/cgi-bin/amigo/browse.cgi). We further added to the BEHS gene set, genes associated with autism spectrum disorder (Iossifov *et al.*, 2014; Yuen *et al.*, 2015), schizophrenia (Carrera *et al.*, 2009; Li *et al.*, 2015; Srinivasan *et al.*, 2015), major depression (Cagliani *et al.*, 2009), and finally the OMIM database (https://www.omim.org) was also consulted for psychopathology associated genes (*i.e.* schizophrenia, major depressive disorder, autism spectrum disorder, asperger syndrome, attention-deficit disorder, antisocial behavior, and obsessive-compulsive disorder) expanding our total dataset. We then excluded genes that were not available for Neanderthal exome analysis, making for a final count of 1,780 genes in IMMS and 278 in BEHS, with a total of 17,246 analyzed genes including target (IMMS or BEHS, accordingly) and control genes.

### Genetic datasets

The high quality exomes of three Neanderthals were retrieved from the Max Planck Institute database (http://cdna.eva.mpg.de/neandertal/exomes/; Castellano *et al.*, 2014). Modern human exome data were obtained from phase 3 of the 1000 Genomes Project (The 1000 Genomes Project Consortium, 2015). To avoid any confounding effects due to interbreeding among archaic humans and modern Eurasians (as reported by Green *et al.*, 2010; Reich *et al.*, 2010; Condemi *et al.*, 2013) in our analysis of balancing selection, we used only Yoruba genomes, as they are assumed to have no admixture history with Neanderthals or Denisovans. We included only autosomal single nucleotide biallelic loci, therefore excluding insertions and deletions (InDels), polymorphic sites with more than two alleles, variants on the sex chromosomes and mitochondrial variants. Only loci found to be heterozygous in the Neanderthal exomes were considered for shared and non-shared polymorphisms; this observed heterozygosity at the individual level was assumed to reflect population-wide polymorphism. Regarding the Yoruba (YRI) sample of 108 individuals, we only considered polymorphisms that both ancestral and derived alleles were segregating in this population. Importantly, as these Neanderthal samples from Croatia and Spain dated to more than 40 Kya (Castellano *et al.*, 2014), we do not expect that any Neanderthal polymorphism is a result of modern human introgression.

Due to the spontaneous deamination of 5-methylcytosine, methylated CpG sites are more prone to mutation than other sites, which raises the probability of allelic identity by state rather than by descent (Azevedo *et al.*, 2015). Because of this, we performed all analyses both including and excluding SNPs located within putatively methylated CpG sites (similarly to Leffler *et al.*, 2013).

The SIFT4G Software (Ng and Henikoff, 2003; Vaser *et al.*, 2016) was used to classify SNPs into synonymous vs. nonsynonymous substitutions and to perform a phenotype prediction for the disruption effect of mutations, allied with known references in literature. Polymorphisms within untranslated regions (UTRs) were excluded from all further analyses.

### Evaluating the excess of trans-SNPs in gene sets

We searched in each gene for polymorphisms shared between Neanderthal and Yoruba exomes (*i.e*. trans-SNPs). We then compared the number of trans-SNPs in each one of the two target gene sets (IMMS and IBHS) to that of the 10,000 gene sets made according to random permutations of all remaining human genes for which Neanderthal exome sequences were available (total of 17,246 genes). In doing so, we always removed genes already accounted for in the target gene set accordingly. For instance, IMMS has 1,780 genes in its dataset, therefore 10,000 random gene sets were built using 15,466 genes as control. Each of the 10,000 random gene sets consisted of as many genes as the target gene set it was simulating, namely 1,780 genes for the comparison to IMMS and 278 to BEHS. The rationale behind the construction of random gene sets, was to generate a null genomic distribution for trans-SNPs, against which each target gene set was then tested. Statistical significance was determined by assessing the deviation in the number of trans-SNPs in the target gene sets in comparison to the background genomic distribution of trans-SNPs generated with the random gene sets. The bash script was used to run this analysis (https://github.com/cegamorim/excess_transSNPs).

Because all loci in each genome were subject to the same demographic history, this approach implicitly controls for demography. However, it does not automatically control for possible effects of background selection, varying mutation rates across sites, and gene size. These effects are known to affect genetic diversity and may therefore bias our results. To control for these effects, genes in the control sets were matched, on a gene-by-gene basis, to those in the IMMS and BEHS target gene sets for background selection, gene length and GC content as follows: Background selection was measured with the B statistic developed by Mcvicker *et al.* (2009), which was computed based on the expected reduction in nucleotide diversity at a neutral site due to purifying selection at other sites, as a function of recombination rates, selected site locations, deleterious mutation rate, and the distribution of selection strengths, and indicates the expected fraction of neutral diversity that is present at a given site. A value close to 0 represents near complete removal of diversity as a result of selection, while a value close to 1 indicates that selection has had little effect on diversity (McVicker *et al.*, 2009). To be matched with a target gene, the value of B for a control gene needed to be within 0.1 units of the value of B for the target gene. Gene size was measured as total exonic length, in accordance with the USCS build hg19 refGene table (https://genome.ucsc.edu/). To be matched, the length of target and control genes needed to be within 400 bp of each other. GC content was calculated considering the gene coordinates described in the refGene table of UCSC build hg19. In a first step, we used BEDTools (Quinlan and Hall 2010) to extract the coding exon sequence based on these coordinates, and then used in-house scripts to determine the GC percentage. To be matched, the GC percentages of target and control genes needed to be within 5% of each other. The criterion for thresholds applied was chosen after many trials where at least one control gene in the exome was found for at least 98% of the target genes in IMMS and BEHS datasets. Those target genes that could not be matched to at least one control gene in the exome were excluded from all further analyses in both the target and control gene sets (Table S2).

All data were handled with vcftools 0.1.13 (Danecek *et al.*, 2011) and bcftools, as well as using in- house Python and bash scripts. Plots and analyses were implemented in the R environment (www.r- project.org).

### Population genetics analyses

In addition to the analysis of trans-SNPs, we considered the classical population genetics statistics Tajima’s *D* and *Fst* as potential markers of balancing selection, indicated by an excess of polymorphisms with low population differentiation. While shared polymorphisms can detect long-term balancing selection, Tajima’s *D* highlights regions with an excess of (not necessarily shared) polymorphisms, due to balancing selection or population size change. On the other hand, *Fst* estimates genetic differentiation among populations. Both, Tajima’s *D* and *Fst* are of interest, since together they indicate potential targets for balancing selection over an intermediate time span, as evaluated using the interval between 0.4 N*e* and 4 N*e* (Fijarczyk and Babik, 2015). Tajima’s *D* for the YRI population and intercontinental *Fst* scores (Global *Fst*) for Yoruba versus Europeans (CEU) and Asians (ASN) were obtained from the 1000 Genomes Selection Browser 1.0 (http://hsb.upf.edu/)(Pybus *et al.*, 2014). It should be stressed that, despite the use of just *H. sapiens* populations, the polymorphisms selected were those shared with Neanderthals. Negative *Fst* values were interpreted as 0. Furthermore, since intermediate allele frequencies are a hallmark of balancing selection (Nielsen *et al.*, 2009), we retrieved the average heterozygosity and standard error for all trans-SNPs from the dbSNP database (https://www.ncbi.nlm.nih.gov/projects/SNP/). A threshold for *Fst* of 0.04, as used by Cagliani *et al.* (2013) to represent low values for *Fst* among human populations, and an average heterozygosity greater than 0.400 (as employed by Pakstis *et al.*, 2010) were considered to flag up SNPs potentially affected by balancing selection.

All relevant SNPs identified in our analyses were queried for known associations with psychiatric disease using the GWAS catalog implemented in the UCSC Table Browser and the available literature.

## Results

We retrieved 22,832 heterozygous sites from the Neanderthal exome (Castellano *et al.*, 2014), which 4,117 were trans-SNPs (Table S3) found within 2,519 genes. Here trans-SNPs are defined as heterozygous sites in Neanderthals that were also polymorphic in modern humans, represented by Yoruba people, according to the 1000 Genomes phase 3 data (The 1000 Genomes Project Consortium, 2015). We note that when *loci* located within CpG sites are included the numbers of such polymorphisms and genes almost doubles (Table S4), which is expected due to the high mutability of such sites (Kong *et al.*, 2012), some of which will potentially not be trans-SNPs but only identical by state.

We sought to evaluate if gene sets related to the immune system (IMMS; 1,754 genes) and behavior (BEHS; 271 genes) were enriched for these trans-SNPs in comparison to the genome as a whole. To do so, we built and permutated 10,000 random sets of genes to be compared to these, by matching each gene in these two sets of genes to others in the genome, controlling for background selection, gene size, and mutation rate (see Material and Methods), since these factors are known to affect the number of polymorphisms in each gene. Implicitly we were also controlling for demography, since we were comparing target genes to others in the same genome, which were therefore subject to the same demographic history. Moreover, because there is no evidence up to date of interbreeding between archaic and modern humans before *H. sapiens* migrated out of Africa, we used Yoruba samples to control for the effect of archaic introgression (Green *et al.*, 2010; Reich *et al.*, 2010; Meyer *et al.*, 2012). Below we describe our findings using this approach for each gene set individually (IMMS and BEHS).

### Signals of balancing selection in the Immune System

**Figure 1:**
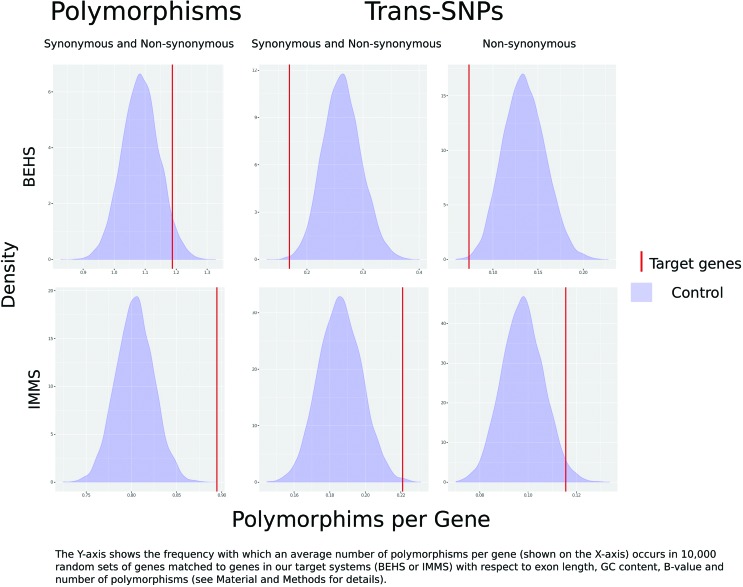
- Density distribution of the average number of polymorphisms per gene observed for random sets genes (blue shade) in the Neanderthal samples matched to those included in the immune system (IMMS) and behavioral system (BEHS) target gene sets (red bars).

We identified 547 trans-SNPs in IMMS genes. This number was significantly higher than the null distribution for trans-SNP observed in the 10,000 random permutations of genes from the control set (p-value= 0.016); Table 1, Figure 1 and Figure S1; see Methods). This pattern remains even if we exclude from the analysis the CpG transitions, which are known to present a higher mutation rate (Table S3). This pattern is borderline significant when considering only shared non- synonymous substitutions (297 polymorphisms; p-value = 0.05). These results suggest that in the genus *Homo*, genes underlying immune system function are more likely than non-immune-genes to maintain long- term shared polymorphisms, possibly through balancing selection. Additionally, we found that Neanderthals IMMS genes harbor more heterozygous sites (2,024 polymorphisms; shared or not with modern humans) than the random sets generated by permutation (p-value < 0.001; Figure 1, column “Polymorphisms”; Table S5). Many of these loci have SNP ID numbers (rs) and have thus been found to be at least biallelic in a modern human population. Due to the known hybridization between Neanderthals and some non-African *H. sapiens* populations, it is difficult to determine whether they represent polymorphisms shared since their split from the common ancestral; nevertheless, these findings reinforce that genes of the immune system maintain a high level of heterozygosity in the genus *Homo*.

**Table 1:** Percentiles of the distribution of the mean values of polymorphism per gene in 10 000 random combinations simulating each of the proposed target gene systems (IMMS and BEHS)^1^.

Percentile		IMMS			BEHS	
%	SNPs	Trans-SNPs NonSyn	Trans-SNPs	SNPs	Trans-SNPs NonSyn	Trans-SNPs
5	0.769932	0.083713	0.166287	0.988971	*0.099265*	*0.209559*
10	0.777335	0.086560	0.170273	1.007350	0.106618	0.220588
15	0.782375	0.088838	0.173121	1.022060	0.110294	0.227941
20	0.786446	0.090547	0.175399	1.036760	0.113971	0.235294
25	0.789863	0.091686	0.177677	1.044120	0.117647	0.238971
30	0.792711	0.093394	0.179385	1.055150	0.121324	0.246324
35	0.796128	0.094533	0.181093	1.062500	0.125000	0.250000
40	0.798405	0.095672	0.182802	1.069850	0.128676	0.253676
45	0.801253	0.096811	0.184510	1.077210	0.132353	0.257353
50	0.804100	0.097950	0.185649	1.084560	0.132353	0.261029
55	0.806378	0.098519	0.187358	1.091910	0.136029	0.264706
60	0.809226	0.099658	0.188497	1.099260	0.139706	0.272059
65	0.811503	0.100797	0.190205	1.110290	0.143382	0.275735
70	0.814351	0.101936	0.191913	1.117650	0.147059	0.279412
75	0.817768	0.103645	0.194191	1.125000	0.150735	0.286765
80	0.821185	0.105353	0.196469	1.136030	0.154412	0.290441
85	0.825171	0.106492	0.198178	1.147060	0.158088	0.297794
90	0.830296	0.109339	0.201025	1.161760	0.165441	0.308824
95	0.838269	0.112187	**0.206150**	1.187500	0.172794	0.319853
100	**0.882688**	0.133827	0.231207	1.327210	0.227941	0.400735

1 Values close to the mean number of polymorphism per gene for each of the target gene sets (IMMS and BEHS) are in italic and underlined, while significant values are in bold. Mean values for the IMMS gene set: Total Neanderthal SNPs = 0.894647, non-synonymous trans-SNPs = 0.115604, trans-SNPs = 0.220957. Mean value for the BeHS gene set: Total Neanderthal SNPs = 1.1875, non-synonymous trans-SNPs = 0.07353, trans-SNPs = 0.169118.

Six of the 547 trans-SNPs shared between Neanderthals and Yoruba that we identified in IMMS (rs2240464, rs56318802, rs5899, rs118014438, rs377657111, and rs14178; Table 2) are located within genes that were previously associated with schizophrenia by the Schizophrenia Working Group of the Psychiatric Genomics Consortium (Schizophrenia Working Group of the Psychiatric Genomics Consortium, 2014). It is noteworthy that another two of the trans-SNPs in IMMS are found in genes that have been associated with this disorder according to another study: rs28919579 is located in the *CD4* gene, a locus that has been linked to schizophrenia through an imbalance of CD4 cell subtypes, and rs374886374 is located within the *C4A* gene, a potential *MHC* locus that has recently been associated with schizophrenia (Sekar *et al.*, 2016). C4 is a fundamental element of the classical complement cascade pathway, which rapidly recognizes and eliminates pathogens and cellular debris. In the brain, *C4A* is expressed in neurons and promotes synaptic pruning, which is impaired in schizophrenic patients (Sekar *et al.*, 2016). The possibility that these trans-SNPs have influenced the behavioral plasticity of Neanderthal and modern humans is speculative, potentially a lot so, but these findings suggest that at least part of the common genetic repertoire that links the IMMS with schizophrenia in modern humans has been maintained polymorphic for thousands of years. We note, however, that immune system genes are known to be highly pleiotropic (Cotsapas *et al.*, 2011; Andreassen *et al.*, 2015; Wang *et al.*, 2015), and this picture may also be true for other traits besides those related to psychiatric disorders. Still, we identified another 60 trans-SNPs located within these 108 loci associated with schizophrenia (Table 2), but that were neither part of our set for IMMS nor BEHS, suggesting that other systems may have trans-SNPs in pleiotropy with schizophrenia.

**Table 2:** Trans-SNPs within loci associated with schizophrenia according to the Schizophrenia Working Group of the Psychiatric Genomics Consortium (2014).

Gene name	Chromosome	Position	ID(rs)	Ancestral allele	Derived allele
*RERE*	1	8419873	rs376434590	T	C
*RERE*	1	8419874	novel	T	G
*RERE*	1	8421203	rs13596	T	C
*BRINP2*	1	177247854	rs31764431	C	G
*CYP26B1*	2	72361960	rs2241057	A	G
*MARS2*	2	198570253	rs200490327	G	A
*GIGYF2*	2	233712227	rs7563724	A	G
*STAB1*	3	52546872	rs150311081	G	A
*STAB1*	3	52548136	rs74491782	T	C
*PBRM1*	3	52643685	rs3755806	T	C
*ITIH4*	3	52850999	rs2535621	A	G
*ITIH4*	3	52863155	rs150181495	G	A
*MUSTN1*	3	52867718	rs2276820	C	T
*ATXN7*	3	63982082	rs3774729	G	A
*KDM3B*	5	137754695	rs7726234	T	C
*DND1*	5	140052271	rs2563333	A	G
*PCDHA1*	5	140168070	rs2240696	A	G
*PCDHA3*	5	140182101	rs7701755	G	T
*PCDHA4*	5	140188383	rs2337987	T	G
*PCDHA4*	5	140188401	rs2879086	A	G
*PCDHA8*	5	140221139	rs3756331	G	A
*PCDHA8*	5	140221195	rs199713478	G	C
*GPX5*	6	28497245	novel	C	T
*GPX5*	6	28497279	rs60523386	G	A
*SCAND3*	6	28541036	novel	C	T
*SCAND3*	6	28543089	rs41270593	G	T
***SRPK2***	**7**	**104782888**	**rs2240464**	**A**	**T**
***SRPK2***	**7**	**104844229**	**rs56318802**	**C**	**T**
*DGKZ*	11	46387868	rs1317826	A	G
*DGKZ*	11	46393138	novel	C	T
***F2***	**11**	**46747662**	**rs5899**	**C**	**T**
*ZDHHC5*	11	57463496	rs140343860	C	T
*ZDHHC5*	11	57466653	novel	G	A
***STAT6***	**12**	**57499258**	**rs118014438**	**C**	**T**
*LRP1*	12	57548364	rs34574998	T	C
*LRP1*	12	57575070	rs199672493	C	T
*LRP1*	12	57592090	rs370217380	G	A
*NXPH4*	12	57619362	rs10783816	G	A
*PITPNM2*	12	123480138	novel	C	T
*CDK2AP1*	12	123749780	rs150530930	G	A
*XRCC3*	14	104165239	novel	G	A
*XRCC3*	14	104169630	rs138987760	C	T
*PLCB2*	15	40590134	rs2229690	G	A
*PLCB2*	15	40594191	rs373064934	A	G
*ADAMTSL3*	15	84706461	rs950169	C	T
***FES***	**15**	**91428302**	**rs377657111**	**C**	**T**
*THAP11*	16	67876823	rs28647874	A	G
*THAP11*	16	67876826	rs3982383	G	A
*THAP11*	16	67876835	rs151159352	G	A
*THAP11*	16	67876844	novel	G	A
*CTRL*	16	67964203	rs1134760	T	C
***PSMB10***	**16**	**67969531**	**rs14178**	**A**	**G**
*SLC12A4*	16	67979051	rs373093291	G	A
*SLC12A4*	16	67980969	rs11860125	G	C
*PLA2G15*	16	68293320	rs3743739	T	C
*SMG6*	17	2203025	rs1885987	T	G
*SMG6*	17	2203356	rs35172468	C	G
*GID4*	17	17948475	rs2955355	G	A
*MYO15A*	17	18022235	rs200234990	C	T
*MYO15A*	17	18023897	rs2955365	G	A
*TSSK6*	19	19625547	rs7250893	A	G
*ZNF536*	19	31038940	rs199936097	G	A
*ZNF536*	19	31038995	rs1469705	T	C
*ACTR5*	20	37377139	rs2254105	C	T
*L3MBTL2*	22	41610024	rs139451	G	A

Loci are sorted by chromosome position. Genes in bold are those belonging to the IMMS target gene set. Genes shaded in gray are those considered as *Credible causal schizophrenia SNPs* (sets of SNPs that are 99% likely to contain the causal variants) according to the Schizophrenia Working Group of the Psychiatric Genomics Consortium (2014).

### Potential signals of balancing selection in Behavior System

No excess of trans-SNPs (shared polymorphisms) was found in BEHS genes (51 SNPs in 41 genes; Table 3; Figure 1 and Figure S1). Likewise, and in contrast to the immune system, the number of heterozygous loci found in Neanderthals, shared and non-shared with Yoruba, corresponds to that expected by chance (p-value = 0.1; Figure 1, column “Polymorphisms”). Additionally, Tajima’s D was not significant for positive values (Table 3). In this regard, significant positive values for Tajima’s D would reflect more pairwise differences than segregating sites due to the increased diversity of the region surrounding the selected site, indicating old balancing selection (Nordborg *et al.*, 1996). On the other hand, we found low *Fst* and high average heterozygosity, both important indicators of potential signals of balancing selection, in five trans-SNPs (rs11176013, rs12628, rs310617, rs438042, and rs362331 (Table 3) shared between Neanderthals and Yoruba. All of these SNPs are associated with psychiatric and neurodevelopmental disorders, including schizophrenia. For instance, rs438042, located near the intron/exon boundary of *THBS4* exon 3, is associated with Alzheimer Disease and might be important for splicing, since *THBS4* acts in inflammatory responses and synaptogenesis (Cagliani *et al.*, 2013; Cocchi *et al.*, 2015). Another trans-SNP identified in the BEHS gene set, rs310617, located in the *EEF1A* gene, has been found to be heterozygous in the Denisova specimen. Although the specific role of rs310617 is not known, other substitutions in this gene have been associated with severe intellectual disability and epileptic encephalopathy (Nakajima *et al.*, 2015; Inui *et al.*, 2016).

**Table 3:** Trans-species polymorphisms identified in genes of the BEHS gene set.

Gene	SNP	Average heterozygosity	Function	*Fst*	Tajima’ *D*	Phenotype	Reference1	Denisova heterozygote
***ALS2***	rs3219156	0.185 +/- 0.240	missense	0.1642	-0.558111591	Amyotrophic lateral sclerosis/parkinsonism-dementia complex	Tomiyama *et al.,*2008	NO
***ALS2***	rs3219168	0.158 +/- 0.233	synonymous	0.1191	-0.093457107	Amyotrophic lateral sclerosis	Kress *et al.,*2005	NO
***ANK2***	rs33966911	0.104 +/- 0.203	synonymous	0.1239	-0.750076422	NA	NA	NO
***ANKRD11***	rs72821356	0.058 +/- 0.160	synonymous	0.0255	-0.931794107	KBG syndrome (OMIM 611192)	NA	NO
***ANKRD11***	rs60520302	0.072 +/- 0.176	missense	0.0019	-0.931794107	KBG syndrome (OMIM 611192)	NA	NO
***APOL2***	rs118097350	0.006 +/- 0.054	missense	0.0005	0.301905912	NA	NA	NO
***BCL11A***	rs7569946	0.282 +/- 0.248	synonymous	0.0742	-0.928248907	Fetal Hemoglobin Level	Bauer *et al.,*2013	NO
***CACNA2D3***	rs17054785	0.181 +/- 0.240	synonymous	0.0766	-0.12464999	NA	NA	NO
***CCDC108***	rs13403802	0.028 +/- 0.115	missense	0.0467	-0.365570142	NA	NA	NO
***CHD1***	rs161941	0.417 +/- 0.186	Synonymous	0.3542	-0.774153927	NA	NA	NO
***CHI3L1***	rs140857184	0.013 +/- 0.080	missense	0.0346	-0.860993564	NA	NA	NO
***CHL1***	rs116261368	0.028 +/- 0.115	missense	0.0409	-0.478853152	NA	NA	NO
***CHL1***	rs2272522	0.428 +- /0.175	missense	0.133	-0.478853152	Schizophrenia	Shaltout *et al.,*2013	NO
***CTNS***	rs161400	0.269 +/- 0.249	missense	0.6469	0.188849119	Nephropatic cystinosis	Shahkarami *et al.,*2013	NO
***CTNS***	rs77453839	0.130 +/- 0.219	synonymous	0.2947	0.383526916	Nephropatic cystinosis	Shahkarami *et al.,*2013	NO
***DBH***	rs1108580	0.490 +/- 0.069	synonymous	0.2628	0.111473168	Schizophrenia, TDAH, cocaine dependence, fetal growth, bipolar disorder	Shaltout *et al.,*2013	NO
***DISC1***	rs55795950	0.004 +/- 0.042	missense	0.0094	-0.209468866	Schizophrenia	Khan *et al.,*2013	NO
***DRD5***	rs184288806	0.002 +/- 0.032	synonymous	NA	NA	NA	NA	NO
***EEF1A2***	rs310617	0.483 +/- 0.090	synonymous	0.0102	0.516242391	Mental retardation, Epilectic encephalopathy (OMIM 602959)	NA	YES
***EIF4EBP2***	rs3750767	0.029 +/- 0.117	synonymous	0.0332	-0.133346514	NA	NA	NO
***GPR153***	rs140518856	0.005 +/- 0.049	synonymous	NA	NA	NA	NA	NO
***GRM5***	rs2306153	0.091 +/- 0.193	synonymous	0.0516	-0.65655565	NA	NA	NO
***HOXB8***	rs45441492	0.114 +/- 0.210	synonymous	0.1385	-0.957471816	NA	NA	NO
***HRAS***	rs12628	0.418 +/- 0.185	synonymous	0.0217	-0.20012551	Costello syndrome	Gripp *et al.,*2011	NO
***HTT***	rs363125	0.307 +/- 0.243	missense	0.3302	-0.83813619	Huntington disease (OMIM 613004)	Kay *et al.,*2014	NO
***HTT***	rs362331	0.493 +/- 0.058	missense	0.0336	-0.718349527	Huntington disease (OMIM 613004)	Lombardi *et al.,*2009	NO
***HTT***	rs140124504	0.002 +/- 0.032	synonymous	NA	NA	Huntington disease (OMIM 613004)	NA	NO
***HTT***	rs138489139	0.002 +/- 0.035	synonymous	NA	NA	Huntington disease (OMIM 613004)	NA	NO
***KDM6B***	rs11078709	0.490 +/- 0.070	synonymous	0.3901	-1.068036831	NA	NA	NO
***KLF12***	rs77377545	0.019 +/- 0.096	synonymous	0.026	-0.747818207	Panic disorder syndrome 1 (OMIM 167870)	NA	NO
***LRRK2***	rs11176013	0.485 +/- 0.084	synonymous	0.0246	0.777713125	Parkinson disease 8 (OMIM 609007)	Mata *et al.,*2006	NO
***LRRTM1***	rs6733871	0.465 +/- 0.128	missense	0.1323	-1.03399393	Schizophrenia	Ludwig *et al.,*2009	NO
***MC4R***	rs2229616	0.032 +/- 0.122	missense	0.01	-1.339833048	Obesity	Heid *et al.,*2008	NO
***MKKS***	rs17852625	0.310 +/- 0.243	synonymous	0.0393	0.147132768	Obesity	Rouskas *et al.,*2008	NO
***MKKS***	rs16991547	0.363 +/- 0.223	synonymous	0.1067	0.147132768	NA	NA	NO
***NMUR2***	rs4958532	0.215 +/- 0.248	missense	0.1464	-0.701022375	NA	NA	NO
***OR4C46***	rs11246606	0.363 +/- 0.223	missense	-0.0025	-1.30558338	NA	NA	NO
***PAK7***	rs55773719	0.013 +/- 0.081	synonymous	0.0145	-0.339650099	NA	NA	NO
***PHF2***	rs56134753	0.040 +/- 0.135	synonymous	0.0199	-0.264535787	NA	NA	NO
***PLCL2***	rs7653834	0.496 +/- 0.047	synonymous	0.054	-0.63849307	NA	NA	NO
***PRODH***	rs139903009	0.004 +/- 0.047	missense	0.0005	0.292869235	Schizophrenia (OMIM 606810)	NA	NO
***PRODH***	rs4819756	0.342 +/- 0.232	missense	0.3182	0.309595729	Schizophrenia	Kempf *et al.,*2008 Ota *et al.,*2014	NO
***PRODH***	rs1808320	0.393 +/- 0.205	synonymous	0.1409	0.369538366	Autism, Schizophrenia	autismkb.cbi.pku.edu.cn; Prata *et al.,*2006	NO
***RGS12***	rs80251844	0.088 +/- 0.191	missense	0.1524	-0.829372734	NA	NA	NO
***RGS12***	rs147416450	0.002 +/- 0.028	missense	NA	NA	NA	NA	NO
***RIMS1***	rs77121218	0.019 +/- 0.097	synonymous	0.0092	-1.576071446	Cone-rod dystrophy (OMIM 606629)	NA	YES
***SCN9A***	rs4369876	0.064 +/- 0.167	missense	0.0735	-0.16967483	Basal Pain Sensitivity	Duan *et al.,*2015	NO
***SHANK1***	rs3745521	0.405 +/- 0.196	missense	0.2752	-0.048981111	Specifc language impairment (OMIM 606712); Mental retardation (OMIM 611097)	NA	NO
***TCF3***	rs11882821	0.033 +/- 0.123	synonymous	0.0467	-1.767706279	NA	NA	NO
***TEKT5***	rs148185751	0.005 +/- 0.051	missense	0.0005	0.183653042	NA	NA	NO
***THBS4***	rs438042	0.490 +/- 0.069	synonymous	0.0334	0.252034645	Alzheimer Disease	Cagliani *et al.,*2013	NO

## Discussion

The evolutionary history of hominins is characterized by several notable and peculiar features. Of particular importance to the successful evolutionary trajectory of the genus *Homo* was the emergence of a large brain capable of sustaining complex and plastic adaptive behaviors, as well as cognitive skills. In recent years, a growing body of research has countered the notion that the Neanderthals were devoid of symbolism and presented lower cognitive abilities and less sophisticated behavior than the early *H. sapiens* of the Paleolithic (Akazawa *et al.*, 1993; Zilhão and Trinkaus, 2002; Zilhão *et al.*, 2010; Pike *et al.*, 2012; Rendu *et al.*, 2013). This is in line with the findings of recent genetic and paleoneurological research. For instance, research by Mounier *et al.* (2016) and Ponce-de-León *et al.* (2016) revealed strong similarities between modern humans and Neanderthals in both endocranial anatomy and general brain development, while a study of 162 genes related to cognition by Paixão-Côrtes *et al.* (2013) identified a genetic repertoire shared between extinct archaic humans and modern humans. Assuming that the use of cognitive skills and complex behavior as an adaptive strategy represent a central element of the human evolutionary trajectory (Cagliani *et al.*, 2009; Schaschl *et al.*, 2015; Taub and Page, 2016), we sought to evaluate whether natural selection, particularly in the form of balancing selection, played any role in the evolution of genes potentially related to human behavior. Examples of a role for balancing selection in behavioral plasticity have been reported from primates (Babb *et al.*, 2010; Claw *et al.*, 2010; Dobson and Brent, 2013; Goto *et al.*, 2016; Taub and Page, 2016), rodents (Lonn *et al.*, 2017) and even arthropods (Fitzpatrick *et al.*, 2007). To detect balanced polymorphisms in genes related to behavior, we implemented an approach based on the search for an excess of shared polymorphisms (trans-SNPs) between archaic and modern humans, in comparison to the genome as a whole.

Our analyses revealed no excess of trans-SNPs in genes known to underlie behavioral traits (captured in our BEHS gene set, see Material and Methods). These findings are consistent with the current knowledge about the genetics underlying *H. sapiens* brain function. Genes expressed in the brain have a large number of functions and the interactions between them are complex (giving rise to basal and specific behavioral phenotypes). Therefore, these genes are subject to functional constraint (Nielsen *et al.*, 2009). Recently, Aggarwala and Voight (2016) developed the concept of genic tolerance to assess the probability of nucleotide substitution in the human genome, based on factors such as population history and selection, among others. In their analysis, genes playing a role in neurodevelopmental and psychiatric disorders were found to have a strong genic intolerance to nucleotide substitution. In the context of functional complexity and constraint, relatively few, key genetic changes can lead to larger effects on certain phenotypes, in response to a specific selective pressure, while at the same time maintaining original functions. Among the results of our analyses, five trans-SNPs located within our set of BEHS (rs11176013, rs12628, rs310617, rs438042, and rs362331; Table 3) presented high average heterozygosity and low *Fst* values, suggesting a homogeneous distribution of both alleles between populations. These results imply that balancing selection did not have a significant role in the evolution of genes implicated in human behavior as a whole*,* but may have been important for the evolution of particular genes within this set. Alternatively, our approach may not have had enough statistical power to detect the effect of balancing selection on the evolution of human behavior. That could be the case if frequency dependent selection, rather than overdominance, was the main mode of balancing selection, since our test is best suited to detect overdominance. Alternatively, the signals for balancing selection in the BEHS set may significantly pre- or postdate the evolutionary split between Neanderthals and modern humans.

Another possibility that deserves to be discussed in the light of our results comes from recent findings suggesting an association between behavioral traits and genes previously implicated in the immune response (Power *et al.*, 2015; Sekar *et al.*, 2016). Through our inter-*Homo* exonic trans-SNP approach, we found that genes underlying immune function (found in the IMMS gene set) contain more ancestral polymorphisms than expected by chance in both Neanderthal and modern humans. These genes have played an important immunological defense role during speciation and migration of the genus *Homo* in a probable similar context of their hominin common ancestral. Our results support the idea that the variability of immune genes is both a target and an outcome of balancing selection (Grimsley *et al.*, 1998; Ségurel *et al.*, 2013). Interestingly, and perhaps surprisingly, several studies have revealed a connection between the genetics of the immune system and human behavior. For instance, some of the strongest genetic associations found for schizophrenia at the population level involve variation in the immune system loci (Schizophrenia Working Group of the Psychiatric Genomics Consortium, 2014; Sekar *et al.*, 2016). It has been suggested that some proteins of the immune system work to promote synaptic pruning (Sekar *et al.*, 2016), which is impaired in schizophrenic patients (Lips *et al.*, 2012). Other mechanisms involved both in the etiology of schizophrenia and in the immune system have also been suggested, including deviant immune responses (Pandarakalam 2013). Some of the IMMS trans-SNPs identified here have previously been found to be associated with schizophrenia in modern humans (rs2240464, rs56318802, rs5899, rs118014438, rs377657111, rs14178, Table 2). Furthermore, the trans-SNP rs374886374 is located in the gene *C4A,* at the *MHC* locus, which has recently also been associated with schizophrenia in a landmark study using several Psychiatric Genomics Consortium cohorts (Sekar *et al.*, 2016; nearly 65,000 individuals; *p-*value < 10-8).

Schizophrenia is known to have a high heritability of around 60-80%, and interestingly, it is frequently contextualized in hypotheses that attempt to explain the evolution of modern human complex behavior (Srinivasan *et al.*, 2015). One hypothesis aiming to reconcile the relatively high prevalence (~0.5 to 1%) of this disorder across human populations with its negative effect on fitness is that balancing selection is maintaining several alleles at loci contributing to creative thinking, a trait that increases fitness. Under unfavorable circumstances, however, the same alleles are thought to increase vulnerability to psychiatric disorders, including schizophrenia (Power *et al.*, 2015; Srinivasan *et al.*, 2015). Our findings contribute to this hypothesis and suggest that some components of the immune genetic repertoire that were maintained polymorphic in both archaic and modern humans could have indirectly influenced the evolution of human behavior. This would represent an extraordinary case of evolutionary co-option, in which IMMS genes under balancing selection harbor ancestral adaptive polymorphisms related to the behavioral plasticity of the genus *Homo*. Allied to our conclusion, recent studies have contributed to unveil the physiological process of autoimmunity in cognition, being proposed as the driving force of cognitive evolution in genus *Homo* (Nataf, 2017).

Finally, a range of other molecular and biological processes certainly play an important role in the evolution of the behavioral plasticity characteristic of *Homo* species, such as gene regulation and epigenetic mechanisms. Moreover, beyond the role of the heterozygote advantage in maintaining these polymorphisms, other forms of natural selection (frequency-dependent, directional, etc.) at multiple levels (*i.e*., individual, kin, and/or group levels; Polimeni and Reiss, 2003; Wilson and Hölldobler, 2005; Zhang and Perc, 2016), together with the unequivocal role of culture (Mesoudi, 2016), have shaped and, in the case of our species, continue to shape, human evolution. A full exploration of these topics is well beyond the scope of the present study, which intends only to explore and discuss some genetic and evolutionary pieces of this complex puzzle. Future studies may help to build a more complete picture of the evolution of hominin behavior.

## Supplementary material

The following online material is available for this article:

Figure S1 Density distribution of the average number of polymorphisms per gene.

Table S1 - GO terms used for target genes playing a role in immune and behavioral systems.

Table S2 - Target genes that could not be matched to at least one similar gene in the exome.

Table S3 - Polymorphisms shared between Neanderthals and modern humans.

Table S4 - Polymorphisms shared between Neanderthals and modern humans, including sites within CpG sites.

Table S5 - Percentiles of the distribution of the mean values of polymorphism per gene.
