# Supplementary material for: Searching for ancient balanced polymorphisms shared between Neanderthals and Modern Humans
Source: Genet Mol Biol. 2018 Jan-Mar;41(1):67–81. doi: 10.1590/1678-4685-GMB-2017-0308 (PMC5901502; doi:10.1590/1678-4685-GMB-2017-0308)
Supplement: Supplementary file 1 [file 1415-4757-GMB-41-01-2017-0308-s002.pdf]

Supplementary Material to "Searching for ancient balanced polymorphisms shared between Neanderthals and Modern Humans"

TableS1 - Gene Ontology (GO) terms used for target genes playing a role in immune and behavioral systems.

GO	Description	System
GO:0002118	Aggressive behavior	Behavioral
GO:0002209	Behavioral defense response	Behavioral
GO:0001662	Behavioral fear response	Behavioral
GO:0048266	Behavioral response to pain	Behavioral
GO:0007619	Courtship behavior	Behavioral
GO:0035640/GO:0035641	Exploratory behavior	Behavioral
GO:0008050	Female courtship behavior	Behavioral
GO:0060180	Female mating behavior	Behavioral
GO:0051866	General adaptation syndrome	Behavioral
GO:0007625	Grooming behavior	Behavioral
GO:0002376	Immune system process	Immune
GO:0002121	Inter-male aggressive behavior	Behavioral
GO:0008049	Male courtship behavior	Behavioral
GO:0060179	Male mating behavior	Behavioral
GO:0002125	Maternal aggressive behavior	Behavioral
GO:0042711	Maternal behavior	Behavioral
GO:0007617	Mating behavior	Behavioral
GO:0044705	Multi-organism reproductive behavior	Behavioral
GO:0007621	Negativeregulationoffemalereceptivity	Behavioral
GO:0060746	Parental behavior	Behavioral
GO:2000822	Regulation of behavioral fear response	Behavioral
GO:0045924	Regulation of female receptivity	Behavioral
GO:2000821	Regulation of grooming behavior	Behavioral
GO:0033057	Reproductive Behavior	Behavioral
GO:0044704	Single-organism reproductive behavior	Behavioral
GO:0035176	Social Behavior	Behavioral
