# Supplementary material for: Searching for ancient balanced polymorphisms shared between Neanderthals and Modern Humans
Source: Genet Mol Biol. 2018 Jan-Mar;41(1):67–81. doi: 10.1590/1678-4685-GMB-2017-0308 (PMC5901502; doi:10.1590/1678-4685-GMB-2017-0308)
Supplement: Supplementary file 2 [file 1415-4757-GMB-41-01-2017-0308-s003.pdf]

Supplementary Material to "Searching for ancient balanced polymorphisms shared between Neanderthals and Modern Humans"

Table S2 –Target genes that could not be matched to at least one similar gene in the exome with respect to exon length, B-value, GC content and number of polymorphisms.

Immune System	Behavior System
ACTB	*EIF4G1*
ACTG1	*HTT*
APOB	*SHANK1*[*]
C4B_2	*TRRAP*
C4B	*WDFY3*
CENPE	*KDM6B*
DYNC1H1	
DYNC2H1	
EP300	
HLA-A	
HLA-B	
HLA-C	
HLA-DQA1	
HLA-DQB1	
HLA-DRB1	
HLA-DRB5	
KMT2A	
LYST	
MAP1A	
MUC4	
NBEAL2	
NCOA6	
NOTCH1[*]	
PIK3C2G[*]	
PKHD1L1	
PRKDC	

[*]Genes excluded only from analyses including CpG sites.
