# Supplementary material for: Searching for ancient balanced polymorphisms shared between Neanderthals and Modern Humans
Source: Genet Mol Biol. 2018 Jan-Mar;41(1):67–81. doi: 10.1590/1678-4685-GMB-2017-0308 (PMC5901502; doi:10.1590/1678-4685-GMB-2017-0308)
Supplement: Supplementary file 3 [file 1415-4757-GMB-41-01-2017-0308-s004.pdf]

Supplementary Material to "Searching for ancient balanced polymorphisms shared between Neanderthals and Modern Humans"

Table S3 - General description of polymorphisms shared between Neanderthals* and modern humans.

Description	Number of polymorphisms
Input matching reference assembly	4117
Input matching as coding regions	99.95% (4115 out of 4117)
Tolerated	86.33% (3554 out of 4117)
Deleterious	10.54% (434 out of 4117)
Not Scored	3.13% (129 out of 4117)
Nonsynonymous	53.22% (2191 out of 4117)
Synonymous	46.85% (1929 out of 4117)
NonCoding (UTR)	0.48% (2 out of 4117)
Novel	7.68% (316 out of 4117)
rsID	92.32% (3801 out of 4117)
Genes	2519

* Observed heterozygosity at the individual level was assumed to reflect population-wide polymorphism.
