# Supplementary material for: Searching for ancient balanced polymorphisms shared between Neanderthals and Modern Humans
Source: Genet Mol Biol. 2018 Jan-Mar;41(1):67–81. doi: 10.1590/1678-4685-GMB-2017-0308 (PMC5901502; doi:10.1590/1678-4685-GMB-2017-0308)
Supplement: Supplementary file 4 [file 1415-4757-GMB-41-01-2017-0308-s005.pdf]

Supplementary Material to “Searching for ancient balanced polymorphisms shared between Neanderthals and Modern Humans”

Table S4 - General description of polymorphisms shared between Neanderthals* and modern humans, including sites within CpG sites.

Description	Number of polymorphisms
Input matching reference assembly	8387
Input matching as coding regions	99.9% (8382 out of 8387)
Tolerated	84.49% (7086 out of 8387)
Deleterious	12.72% (1067 out of 8387)
Not Scored	2.79% (234 out of 8387)
Nonsynonymous	49.81% (4175 out of 8382)
Synonymous	50.19% (4207 out of 8382)
NonCoding (UTR) and lincRNA	5.96 x 10-4(5 out of 8387)
Novel	11.42% (958 out of 8387)
rsID	88.58% (7429 out of 8387)
Genes	4900

* Observed heterozygosity at the individual level was assumed to reflect population-wide polymorphism.
