# Supplementary material for: Searching for ancient balanced polymorphisms shared between Neanderthals and Modern Humans
Source: Genet Mol Biol. 2018 Jan-Mar;41(1):67–81. doi: 10.1590/1678-4685-GMB-2017-0308 (PMC5901502; doi:10.1590/1678-4685-GMB-2017-0308)
Supplement: Supplementary file 5 [file 1415-4757-GMB-41-01-2017-0308-s001.pdf]

Supplementary Material to "Searching for ancient balanced polymorphisms shared between Neanderthals and Modern Humans"

Figure S1 - Density distribution of the average number of polymorphisms per gene observed for random sets of genes (blue shade) in the Neanderthal samples matched to those included in the IMMS and BEHS target gene sets (red bars), including genes located in CpG sites.

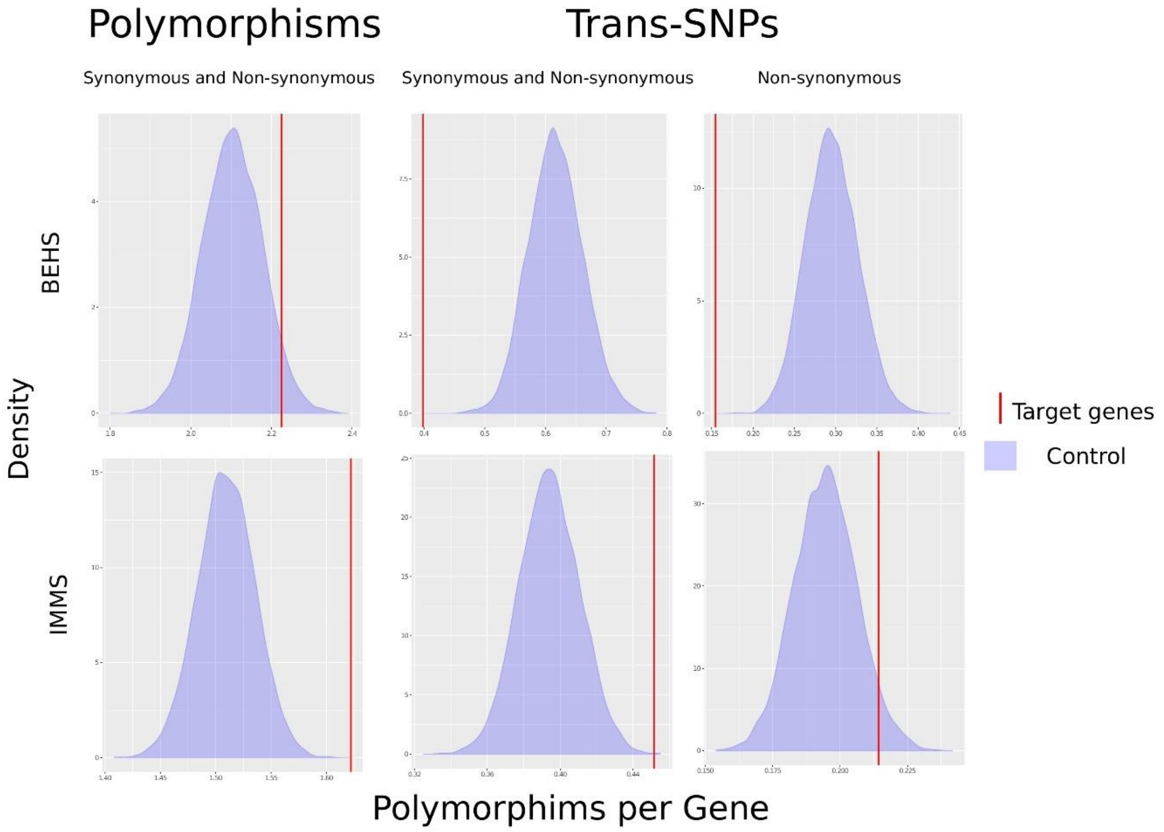

The Y-axis shows the frequency with which an average number of polymorphisms per gene (shown on the X-axis) occurs in 10,000 random sets of genes matched to genes in our target systems (BEHS or IMMS) with respect to exon length, GC content, B-value and number of polymorphisms (see Material and Methods for details).
