# Supplementary material for: Searching for ancient balanced polymorphisms shared between Neanderthals and Modern Humans
Source: Genet Mol Biol. 2018 Jan-Mar;41(1):67–81. doi: 10.1590/1678-4685-GMB-2017-0308 (PMC5901502; doi:10.1590/1678-4685-GMB-2017-0308)
Supplement: Supplementary file 6 [file 1415-4757-GMB-41-01-2017-0308-s006.pdf]

Supplementary Material to "Searching for ancient balanced polymorphisms shared between Neanderthals and Modern Humans"

Table S5 - Percentiles of the distribution of the mean values of polymorphism per gene in 10,000 random combinations simulating each of the proposed target gene systems (IMMS and BEHS), including sites within CpG sites[1].

Percentile	IMMS			BEHS		
%	SNPs	Shared NonSyn	Shared SNPs	SNPs	Shared NonSyn	Shared SNPs
5	1.466360	0.175599	0.367161	1.981550	*0.243542*	*0.546125*
10	1.476050	0.179590	0.372862	2.007380	0.254613	0.560886
15	1.482330	0.182440	0.376853	2.025830	0.261993	0.568266
20	1.487460	0.184721	0.379704	2.040590	0.269373	0.579336
25	1.492020	0.187001	0.382554	2.051660	0.273063	0.586716
30	1.496010	0.188712	0.385405	2.066420	0.276753	0.594096
35	1.499430	0.189852	0.387685	2.073800	0.284133	0.597786
40	1.502850	0.191562	0.389966	2.084870	0.287823	0.605166
45	1.506270	0.193273	0.391676	2.095940	0.291513	0.608856
50	1.509690	0.194413	0.393957	2.103320	0.295203	0.616236
55	1.513110	0.196123	0.396237	2.114390	0.298893	0.619926
60	1.516530	0.197263	0.397948	2.121770	0.302583	0.627306

Percentile	IMMS			BEHS		
65	1.519950	0.198974	0.400228	2.132840	0.306273	0.634686
70	1.523380	0.200684	0.402509	2.143910	0.309963	0.638376
75	1.527370	0.202395	0.405359	2.154980	0.317343	0.645756
80	1.531360	0.204675	0.408210	2.166050	0.321033	0.653137
85	1.536490	0.206956	0.411631	2.180810	0.328413	0.664207
90	1.542817	0.209806	0.416192	2.199260	0.335793	0.675277
95	1.552450	0.213797	0.421352	2.225090	0.346863	0.690037
100	**1.606040**	0.241733	**0.454960**	2.391140	0.439114	0.782288

[1] Values close to the mean number of polymorphism per gene for each of the target gene sets (IMMS and BEHS) are in italic and underlined, while significant values are in bold. Mean values for the IMMS gene set: Shared and non-shared SNPs = 1.62201, Shared non-synonymous polymorphisms = 0.214367, Shared SNPs = 0.451539; Mean values for the BEHS gene set: 2.225090, 0.154982, 0.398524.
